# Stigma toward substance use disorders: a multinational perspective and call for action

**DOI:** 10.3389/fpsyt.2024.1295818

**Published:** 2024-02-01

**Authors:** Samer El Hayek, Wael Foad, Renato de Filippis, Abhishek Ghosh, Nadine Koukach, Aala Mahgoub Mohammed Khier, Sagun Ballav Pant, Vanessa Padilla, Rodrigo Ramalho, Hossameldin Tolba, Mohammadreza Shalbafan

**Affiliations:** ^1^ Medical Department, Erada Center for Treatment and Rehabilitation in Dubai, Dubai, United Arab Emirates; ^2^ Psychiatry Unit, Department of Health Sciences, University Magna Graecia of Catanzaro, Catanzaro, Italy; ^3^ Postgraduate Institute of Medical Education and Research, Chandigarh, India; ^4^ Department of Psychiatry, Institute of Medicine, Tribhuvan University, Kathmandu, Nepal; ^5^ Department of Psychiatry and Behavioral Sciences, University of Miami, Miami, FL, United States; ^6^ Department of Social and Community Health, University of Auckland, Auckland, New Zealand; ^7^ Mental Health Research Center, Psychosocial Health Research Institute (PHRI), Department of Psychiatry, School of Medicine, Iran University of Medical Sciences, Tehran, Iran

**Keywords:** stigma, social stigma, discrimination, addiction, substance use disorder, psychiatry

## 1 Introduction

The stigma surrounding persons living with substance use disorders (SUDs) is a ubiquitous phenomenon that has had detrimental effects on affected individuals, their families, healthcare providers, treatment outcomes, research, policies, and society as a whole (1). Studies suggest that the stigma toward SUDs exceeds that of other mental health conditions (2) and is a common obstacle to help-seeking behavior among individuals with SUDs (3, 4). One systematic review found that healthcare professionals typically hold negative attitudes toward people with SUDs, resulting in patients experiencing reduced empowerment and poorer treatment outcomes (5). As healthcare professionals are often the gatekeepers to treatment, they must be adequately trained and educated about managing SUDs. Medical bodies, educational institutes, and various governmental and non-governmental organizations are recognizing that substance use treatment, policies, and language need to evolve to provide better support for affected individuals (1).

In this opinion piece that brings the wisdom and experience of a multinational group of adult and addiction psychiatrists, we highlight the different sources of stigma faced by individuals with SUDs and how it impacts treatment-seeking and related outcomes. More importantly, we provide recommendations for holistic interventions to address stigma toward addiction and enhance the delivery of optimal care.

## 2 Stigma toward addiction and its impact on treatment

### 2.1 Where does stigma originate from?

Stigma toward SUDs comes from many sources including society, individuals living with SUDs themselves, their families, and healthcare professionals (6). Many factors can play a role in perpetuating this stigma, especially the longstanding societal view of addiction as a personal or moral failure (6, 7). In different societies and cultures, it is not uncommon for the community to label individuals with SUDs as worthless, weak, dangerous, criminals, incapable of holding jobs or forming families, and having deliberately chosen addiction. Some specific stereotypes faced by individuals with SUDs can be related to disadvantaged and vulnerable populations, such as those living in poverty or homelessness, minorities, and those who allow “peer pressure” to influence their drug use (8). In Nepal, individuals with a history of SUD continue to be perceived as lacking moral values, despite undergoing treatment, which can lead to relapse (9). One systematic thematic analysis of Indian newspaper articles explored online media’s attitudes and perceptions toward individuals with SUDs. Results showed that many articles propagated public stigma by using stigmatizing language, identifying people who use drugs with negative and unwanted qualities (10). Similarly, the media in New Zealand had often used terms such as “meth head” and “drug addict” when discussing individuals with SUDs (11). In Italy, schools, universities, and media outlets rarely convey the medical aspects of addictions, and SUDs are typically perceived as vices rather than diseases. Along the same lines, addiction treatment centers tend to be isolated and external to the general hospital setting (12, 13). Criminalizing drug use further fosters public stigma toward individuals with SUDs, through social and economic marginalization. Criminalization also diverts attention from the medical and public health models of addiction to a moral and punitive model. In India, for instance, this translates in the latest Narcotic Drugs and Psychotropic Substances policy limiting the access to evidence-based treatment, such as opioid agonist maintenance treatment and harm reduction strategies, by imposing restrictions on the duration and settings of treatment (14). Similarly, in Nepal, current laws remain harsh and add to the perception that individuals with SUDs are criminals who should be referred to the criminal justice system (15).

Individuals with SUDs and their family members experience significant internalized and affiliate stigma. Across cultures, internalized stigma is correlated with poorer quality of life (16) and delay in seeking treatment (17), especially in the initial stages of the illness (18). The Arab, Iranian, and Indian societies are collectivistic societies where family plays an essential role in fostering support and individuals’ decision-making. Hence, if one family member is affected by a SUD, others would attempt to provide support. However, families would also try hard to shield the affected member from the neighborhood and society to avoid stigma, which can lead to delays in treatment. In general, affiliate stigma tends to prevent families from providing the necessary medical support to their loved ones (19).

Healthcare professionals also hold negative perceptions and implicit biases toward individuals with SUDs. One study among primary care providers in New Zealand found that people who misuse prescription medications can be often stigmatized and offered limited harm reduction interventions (20). A systematic review of studies conducted in Western countries found that negative attitudes of healthcare professionals toward individuals with SUDs are common and contribute to suboptimal care (5). The general attitude of healthcare providers toward people with lived experience of mental illness is not positive in Iran, and attitudes toward those living with SUDs seem to be worse (21, 22). In many countries, including the Arab world and India, little enthusiasm is noted among psychiatry trainees to take up addiction psychiatry as a career option. Minimal exposure during residency, non-availability of evidence-based treatments and limited awareness about them, and significant public stigma might contribute to this. For similar reasons, practicing psychiatrists are sometimes uncomfortable with treating individuals with SUDs and are reluctant to initiate medications, perceiving this population as “challenging” and “difficult”.

### 2.2 How does stigma impact treatment?

Stigma is a negative driver for recovery from addiction. As previously mentioned, individuals who are stigmatized are less willing to engage in or seek medical treatment. Moreover, they may have to deal with associated fear, anger, isolation, trauma, or comorbid mental health disorders. This leads to care avoidance, self-directed early hospital discharge, and hesitance to call medical help or accept transport to a hospital after an overdose, all secondary to the fear of stigma and legal consequences (4). Stigma also impacts treatment availability, with a noted scarcity in the workforce that caters to the needs of individuals with SUDs, insufficiency in the number of treatment centers and available interventions, and limited support groups. Along the same lines, research into the treatment of addictions tends to be less prioritized than that of other mental illnesses. The stigmatizing language used to describe substance use behaviors, individuals with SUDs, and substance use treatment also creates other types of barriers for individuals who are on the road to recovery and re-integration into society, including at the level of their general healthcare, housing, employment, and insurance policies.

In many parts of the world, this translates into inappropriate and potentially hazardous treatment methodologies. For instance, in India, some privately owned rehabilitation centers deliver unethical and punitive treatments that lack evidence- and right-based medical approaches (23). In Iran, families can push their relatives with SUDs for involuntary admissions to “Campus”, which represents mandatory residence places that fail to provide proper treatment. In Nepal, people with SUDs have been traditionally treated out of health systems, in shoddy rehabilitation centers run by people with no experience and no standardized treatment protocol (15). In the Arab world, drug use can still be seen as a form of breakdown, possession by an evil spirit, or shortcoming of individual religious faith; and people may pursue a religious healer instead of a professional mental health practitioner to address this problem (24). But even when individuals seek professional help from psychiatrists or other allied medical health professionals, they can certainly be subject to inaccurate treatment approaches, highlighting the need for improving the awareness of the different stakeholders about the available evidence-based interventions.

## 3 Discussion

There is an urgent need to combat the stigma surrounding SUDs. Research on stigma interventions for providers who treat individuals with SUDs increased in recent years, indicating greater worldwide attention to the negative impact of stigma (25). **Table 1** presents a comprehensive summary of potential interventions and strategies to decrease stigma toward addiction and individuals living with SUDs. By recognizing the enormous challenge that stigma poses to communities, and by revising the words and terms used when discussing matters of addiction and the people living with it, major reforms can occur. Decriminalizing drug use is another pivotal step that can guide the way. This measure not only decreases stigma but also allows for a shift in resources toward prevention and treatment, promoting an approach that prioritizes healing over punishment.

**Table 1:** Strategies to reduce stigma related to addiction and individuals with SUDs.

Introducing training on SUDs in medical and nursing schools.	Promoting destigmatizing language when communicating with people with lived or living experience of addiction, their families, and the community: - Utilize “person-first” language, emphasizing the individual rather than their condition. - Replace terms such as “substance abuser”, “drug abuser”, “addict”, “alcoholic”, “drunk”, “junkie”, and “user” with “person with substance use disorder” and “person in active use of”. - Replace “clean person” and “ex-addict” with “person in recovery” and “person who previously used drugs”. - Replace “stayed clean” with “maintained recovery”. - Replace “habit” with “substance use disorder” and “drug addiction”. - Replace “abuse” with “use” for illicit drugs and “misuse” or “used other than prescribed” for prescription medications. - Replace “clean/dirty” toxicology results with “positive/negative” toxicology results. - Replace “drug offender” with “person arrested for drug use or drug violation”. - Replace “refused” and “non-compliant” with “chose not to at this point”. These changes emphasize that the person “has” a problem, rather than “is” the problem and help avoid negative connotations, punitive attitudes, and individual blame.
Increasing training and engagement in addiction psychiatry during post-graduation psychiatry residency, as well as in other residencies such as emergency medicine and family medicine.	
Encouraging research in the field of SUDs, with a particular focus on generating more evidence for culturally validated and accepted psychosocial treatments for SUDs.	
Making evidence-based treatment widely available and accessible to individuals with SUDs.	
Introducing courses that explain the biological elements and the concept of SUDs in educational bodies, such as schools and universities.	
Developing social media guidelines for reporting individuals with SUDs, using destigmatizing language.	
Implementing social media awareness campaigns to encourage discussions about addiction and help-seeking.	
Educating high-profile individuals and influencers, including religious leaders, on available evidence-based treatments to enable them to provide referrals when encountering individuals with SUDs.	
Increasing stakeholder and community engagement in both research and awareness campaigns to facilitate conversations and raise awareness about the topic.	
Improving the social inclusion of individuals with SUDs through better access to job opportunities and health insurance, among other measures.	
Decriminalizing substance use (excluding possession, sale, or drug dealing).	

On this path of improvement, it is necessary to highlight some of the extensive efforts, campaigns, and work-in-progress initiated to battle the stigma against SUDs. For instance, in the United States, the Substance Abuse and Mental Health Services Administration (SAMHSA) and the National Institute on Drug Abuse (NIDA) continue to make widespread efforts to educate and break the stigma. In Egypt, the Ministry of Health has re-launched its “You Can, Without It” campaign for the fifth year in a row to aid individuals in recovery in avoiding relapse. Erada Center for Treatment and Rehabilitation in Dubai has launched for the past four years the “Masmooh” Campaign, a television/radio initiative that aims to educate the community about the nature of SUDs and promote greater understanding of the role of forgiveness in helping individuals recover. “Masmooh”, which means “permitted”, seeks to promote forgiveness in society to help re-integrate individuals with SUDs into their communities and give them a sense of hope. New Zealand has a strong history of health promotion, prevention, and anti-discrimination advocacy, with various campaigns targeting non-behavioral addictions, such as “Say Yeah, Nah”, “Go the distance”, and ‘Changing Minds’. Worldwide, several countries, governments, institutions, and educational bodies have shared online material to promote SUD-related anti-stigma campaigns and provide training and workshops for policymakers, healthcare professionals, educators, employers, faith leaders, youth, and the public. Equally important, major medical journals have been more keen on publishing about the topic of stigma toward SUDs in a compounded effort to educate the public (26).

Still, more work is needed. We, as psychiatrists, advocate for a stigma-free approach when treating individuals with SUDs. Such an approach includes actively listening to the person’s story, using destigmatizing language (27, 28), avoiding medical jargon, and ensuring to ask every individual how comfortable they feel talking about their substance use and how ready they are to change their use. Treatment should be provided in an individualized manner. Importantly, the approach should be free from judgment and prejudice, building trust by respecting the person’s rights for confidentiality and choice of treatment strategies, and respecting their dignity, beliefs, and culture. Autonomy and freedom to choose or not choose treatment or any particular treatment is essential. While using a motivational interviewing approach, providers should actively work on offering evidence-based treatments, including pharmacological interventions and prompt referral to medication-assisted treatments, individual and group therapies, and referral to peer support groups, such as Alcoholics Anonymous (AA) and Narcotics Anonymous (NA) meetings. Emphasis is on the adoption of a harm-reduction approach, including the provision of a naloxone rescue kit and encouragement of safe injecting practices. This helps to highlight that the problem is substance use-related harm, not the person nor the substance use. Of equal significance is the incorporation of a trauma-informed care approach for all individuals with SUDs (29). Alternatively, using analogies of chronic non-communicable diseases (e.g., hypertension, diabetes mellitus) can be helpful for individuals to understand their condition. Lastly, instilling hope is of utmost importance, through educating people and their families that SUD is a treatable medical condition, not a choice, and that the person is never defined by their drug use. In the end, fostering a more accepting society involves numerous avenues, yet it always starts with grassroots initiatives, such as our language choices in everyday clinical discourse.

## Author contributions

SEH: Conceptualization, Data curation, Investigation, Writing – original draft. WF: Conceptualization, Data curation, Writing – original draft. RdF: Conceptualization, Data curation, Writing – review & editing. AG: Data curation, Writing – review & editing. NK: Data curation, Writing – review & editing. AMMK: Data curation, Writing – review & editing. SP: Data curation, Writing – review & editing. VP: Data curation, Writing – review & editing. RR: Data curation, Writing – review & editing. HT: Data curation, Writing – review & editing. MS: Conceptualization, Data curation, Supervision, Writing – review & editing.
